# Breast and axillary surgery after neoadjuvant systemic treatment – A review of clinical routine recommendations and the latest clinical research^☆^

**DOI:** 10.1016/j.breast.2022.01.008

**Published:** 2022-01-22

**Authors:** André Pfob, Joerg Heil

**Affiliations:** University Breast Unit, Department of Obstetrics and Gynecology, Heidelberg University Hospital, Heidelberg, Germany

**Keywords:** Breast cancer, Neoadjuvant systemic treatment, Pathologic complete response, Surgery, Biopsy, Intelligent VAB

## Abstract

Breast and axillary surgery after neoadjuvant systemic treatment for women with breast cancer has undergone multiple paradigm changes within the past years. In this review, we provide a state-of-the-art overview of breast and axillary surgery after neoadjuvant systemic treatment from both, a clinical routine perspective and a clinical research perspective. For axillary disease, axillary lymph node dissection, sentinel lymph node biopsy, or targeted axillary dissection are nowadays recommended depending on the lymph node status before and after neoadjuvant systemic treatment. For the primary tumor in the breast, breast conserving surgery remains the standard of care. The clinical management of exceptional responders to neoadjuvant systemic treatment is a pressing knowledge gap due to the increasing number of patients who achieve a pathologic complete response to neoadjuvant systemic treatment and for whom surgery may have no therapeutic benefit. Current clinical research evaluates whether less invasive procedures can exclude residual cancer after neoadjuvant systemic treatment as reliably as surgery to possibly omit surgery for those patients in the future.

## Highlights

• Breast and axillary surgery after neoadjuvant systemic treatment has evolved.
• Choice of axillary surgery depends on lymph node status before and after treatment.
• Optimal management of exceptional responders to neoadjuvant treatment is unclear.
• Clinical research aims to reliably exclude residual cancer without surgery.
• For exceptional responders, breast cancer surgery may be omitted in the future.

## 1 Introduction

Over the past 100 years, breast cancer surgery has undergone multiple paradigm changes [1]: While (radical) mastectomy and axillary lymph node dissection (ALND) used to be the standard of care, more tailored and less invasive procedures like breast conserving surgery and sentinel lymph node biopsy (SLNB) embedded into multi-modal therapy concepts (surgery, systemic treatment, and radiation) are nowadays recommended for most women with early-stage breast cancer.

The introduction of neoadjuvant systemic treatment (NST) (chemotherapy and targeted antibody therapy) has long been considered a double-edged sword from a breast surgical perspective. On the one hand, NST often shrinks the tumor before surgery which allows for surgical downstaging and less invasive breast-conserving surgery to spare our patients relevant treatment-associated morbidity [2]. On the other hand, the oncologic safety of less invasive breast conserving surgery and SLNB after NST has been unclear for a long time because they had shown equivalent oncologic safety in an adjuvant therapy setting which may, however, not apply for the neoadjuvant setting [[3], [4], [5]]. However, later studies showed that both, SLNB and breast conserving surgery after NST are not inferior to ALND and mastectomy in terms of oncologic outcomes [[6], [7], [8]]. In fact, the increasing use and improved efficacy of NST is associated with a growing number of patients who do not have any detectible tumor left upon surgery (60–70% for triple-negative and HER2 positive cancers [9,10]) which has led to considerations to forego surgery altogether for those exceptional responders to NST [11,12].

In this review, we provide a state-of-the-art overview of breast and axillary surgery after NST from both, a clinical routine perspective and a clinical research perspective. We will focus on the surgical management of four scenarios (Table 1): patients with nodal positive breast cancer (cN+), patients with nodal negative breast cancer (cN0), patients who undergo breast conserving surgery after NST, and patients with an exceptional response to NST (ypT0, ycN0).

**Table 1:** Clinical scenarios for the surgical management of breast cancer patients after neoadjuvant systemic treatment.

	(Potential) paradigm changes	Oncologic safety – overall survival	Oncologic safety – LRFS	Diagnostic performance (false-negative rate)	Guideline recommendation [13]
1) cN+	ALND - > TAD/SLNB	?	?	<8% [[14], [15], [16]]	SLNB (≥3 removed SLNs) or TAD
2) cN0	SLNB - > no surgery	?	?	<5% (if ypT0) [17]	SLNB
3) cT+	Mastectomy- > Breast conserving surgery	Yes [6]	Yes/No [6]	?	Breast conserving surgery
4) cT+/ycT0	Breast conserving surgery - > no surgery	?	?	0–50% for minimally invasive biopsies [[18], [19], [20], [21], [22], [23], [24], [25]]	Breast conserving surgery
4.1) cN+/0, cT 1–3/ycT0/ycN0	TAD/SLNB/breast conserving surgery - > no breast and axillary surgery	?	?	<5% intelligent vacuum-assisted biopsy [26]	TAD/SLNB/breast conserving surgery

TAD = targeted axillary dissection; LRFS = local recurrence free survival; SLNs = sentinel lymph nodes.

## 2 Axillary surgical management

The axillary surgical management of breast cancer patients has undergone multiple changes since the introduction of NST. In the adjuvant setting, we learned that even leaving some tumor behind (non-sentinel lymph node metastasis for patients who undergo SLNB instead of ALND) does not impair oncologic safety but spares our patients relevant treatment-associated morbidity (i.e. lymphedema, chronic pain, mobility restrictions) [[27], [28], [29]]. In the neoadjuvant setting, however, the diagnostic performance of axillary staging had to be re-assessed. Generally, we can distinguish patients with (histological) nodal negative disease before and after NST (cN0, ycN0), and patients with nodal positive disease before NST (cN+) who remain nodal positive after NST (ycN+) or who convert to nodal negative disease (ycN0). Due to limited diagnostic accuracy of ultrasonography to determine ycN status (about 50% of patients with sonographic ycN0 status present residual axillary disease in the surgical specimen), the key difference lays in the axillary surgical staging of cN + vs. cN0 patients [30]. Over the past years, clinical trials assessed the diagnostic performance of different axillary staging procedures (ALND, SLNB, TAD) for these patients (Table 2).

**Table 2:** Current clinical routine recommendations for axillary surgical management.

	False-negative rate	Survival	Guideline recommendation [13]
cN0	SLNB 7% [31]	?	SLNB
cN+	SLNB 13% [16,[31], [32], [33]] SLNB with ≥3 removed SLNs 8% [16] TAD 2% [[14], [15], [16]]	?	TAD/SLNB (≥3 removed SLNs)
ypN+	–	SLNB inferior compared to ALND [34]	ALND

SLNB = sentinel lymph node biopsy; ALND = axillary lymph node dissection; TAD = targeted axillary dissection; SLN = sentinel lymph nodes.

For patients with initial cN0 status, SLNB after NST is recommended (although, with a false-negative rate 7%, SLNB is no perfect diagnostic procedure in this setting as well) [31].

For patients with cN + status, SLNB initially showed a high risk of leaving tumor behind (false-negative rate >10%) [16,[31], [32], [33]]. However, subsequent studies showed that the diagnostic performance improves with the removal of at least 3 sentinel lymph nodes (FNR 8%) or with TAD (SLNB + removal of clipped nodes, false-negative rate <4%) [[14], [15], [16]]. Thus, SLNB with removal of ≥3 sentinel lymph nodes or TAD is nowadays recommended for patients with cN + disease instead of ALND.

Patients who present with histopathologic residual axillary disease (ypN+) as determined by axillary surgical staging (SLNB or TAD) are recommended completion ALND, as retrospective registry studies suggest that the omission of ALND in case of ypN + status results in inferior survival [34]. However, ongoing trials like the Alliance A011202 trial (NCT01901094) evaluate the feasibility of substituting ALND by extended nodal radiation [35]. Until further evidence is available, the omission of ALND should be considered experimental for patients with ypN + status.

Notably, guideline recommendations for routine axillary surgical management have changed based on the results of diagnostic clinical trials – to date, there is no high-quality evidence on actual (long-term) oncologic outcomes like local control or survival for de-escalated axillary staging following NST. The general assumption is that a false-negative rate of <10% will not translate into impaired oncologic outcomes which, in fact, could be observed for de-escalated axillary staging in the adjuvant setting [27,28,36,37]. However, future research may be necessary to verify this assumption.

Current clinical research in breast cancer axillary staging also focuses on patients with (y)cN0 disease and the question of whether there is a specific group of patients who may not benefit at all from axillary staging and whom we could spare this procedure. A clinical trial (NCT04101851) has just commenced evaluating whether it is oncologically safe to omit SLNB in triple-negative and HER2-positive breast cancer patients with radiologic and pathological complete response in the breast after NST [38]. We know that residual axillary disease is very rare in women with triple-negative or HER2-positive breast cancer and with a complete response to NST in the breast (ypT0) [17]. Thus, the hypothesis is that axillary staging for patients with a pathologic complete response in the breast has no therapeutic benefit as the development of axillary metastasis is driven by the biology of the primary tumor in the breast. The results of the primary endpoint evaluation are expected in 2028.

## 3 Breast surgical management

Not only the axillary but also the breast surgical management of breast cancer patients has been influenced by the introduction of NST. Although breast conserving surgery (followed by radiotherapy) showed equivalent survival compared to mastectomy in the neoadjuvant and adjuvant setting, some evidence suggests that “less surgery” after NST may be associated with higher local recurrence rates [6]. One should consider that the effect observed in this meta-analysis was mainly attributed to two trials from 1983 to 1985 that did not perform any surgery after NST; missing consideration of axillary and margin status may have contributed to this observation as well. More recent evidence suggests that the risk of local recurrence is driven by tumor biology rather than neoadjuvant vs. adjuvant treatment [39]. Thus, breast conserving surgery remains the current standard also in the neoadjuvant setting. Interestingly, the question of whether mastectomy should be de-escalated to breast conserving surgery after NST was informed by oncologic outcome data in contrast to the diagnostic performance data which has led to the de-escalation of axillary surgery over the past years.

The increasing applications and efficacy of NST led to another pressing question in breast surgical care: How should we deal with the growing number of patients who achieve a pathologic complete response to NST (i.e. no residual cancer in the breast and axillary surgical specimen, ypN0, ypT0)? Modern therapy regimens showed pathologic complete response rates of 60–70% for HER2+ and triple-negative breast cancer in large phase III trials and there is growing evidence that also patients with high-proliferative Luminal B breast cancer may benefit from NST [9,10]. As all (local) tumor has already been eradicated by NST, it is unlikely that these patients without histopathological residual cancer benefit from a surgical procedure – in fact, surgery for those exceptional responders to NST may rather be considered as a diagnostic procedure (to reliably exclude residual cancer) and not as a primary therapeutic procedure anymore. Thus, current clinical research tries to identify and develop other, less invasive procedures that can exclude residual cancer after NST as reliably as surgery. If such a diagnostic tool could be identified, we may spare patients without residual cancer after NST invasive surgery.

Several diagnostic tools have been evaluated to exclude residual cancer after NST. We learned that imaging after NST is not accurate enough to replace surgery: ultrasound and mammography show high rates of missed residual cancer (about 20%) and although MRI and PET-CT miss less cancer they show high rates of false-positive findings which restricts the clinical applicability [40,41]. Recently, the use of minimally invasive biopsies after NST to reliably exclude residual cancer has been evaluated (Table 3). Although these biopsies showed promising results in several pilot trials, larger prospective trials failed to confirm a sufficiently high diagnostic accuracy to replace surgery [[18], [19], [20], [21], [22], [23], [24], [25]]. Especially small, heterogeneous responding tumor foci are at risk of being missed by minimally invasive biopsies [18,21]. Recently, a so-called intelligent vacuum-assisted biopsy (intelligent VAB) was developed to address this problem. The intelligent VAB is an artificial intelligence algorithm that uses not only the results of a minimally invasive biopsy after NST but also of contextualizing imaging, patient, and tumor variables to calculate the risk of residual cancer. Such intelligent algorithms have already shown great performance in other medical fields to provide accurate predictions tailored to the individual patient [[42], [43], [44], [45], [46]]. The intelligent vacuum-assisted biopsy showed a promising false-negative rate of 0% to exclude residual cancer in the breast (ypT0) in an external validation set [19]. However, prospective confirmatory evidence that would justify foregoing breast surgery in exceptional responders to NST is still missing.

**Table 3:** Clinical trials evaluating the diagnostic accuracy of minimally invasive biopsies to exclude residual cancer after neoadjuvant systemic treatment.

Clinical trial	Study type	Study details	Sample size	False-negative rate – whole cohort	False-negative rate – subgroup
Heil et al., 2016 [25]	Prospective, single center	Ultrasound- or mammography-guided VAB	n = 50	26% (95% CI, 14–38%)	4.9% (if histopathologically representative biopsy sample; n = 38)
Kuerer et al., 2018 [22]	Prospective, single center	Ultrasound- or mammography-guided VAB or FNA	n = 40	5% (95% CI, 0–24%)	NA
Lee et al., 2020 [23]	Prospective, single center	Ultrasound-guided VAB or CNB	n = 40	31% (95% CI, 14–70%)	0% (if lesion on post-NST MRI ≤0.5 cm, lesion-to-background signal enhancement ratio ≤1.6, and ≥5 biopsy cores; n = 27)
Heil et al., 2020 [18]	Prospective, multi-center	Ultrasound- or mammography-guided VAB	n = 398	18% (95% CI, 13–24%)	0% (for 7-gauge needles; n = 41) 6.2% (if no residual disease in biopsy and on post NST imaging)
van Loevezijn et al., 2020 [21]	Prospective, multi-center	Ultrasound-guided CNB	n = 167	37% (95% CI, 27–49%)	NA
Basik et al., 2020 [20]	Prospective, multi-center		n = 98	50% (95% CI, 33–67%)	NA
Tasoulis et al., 2020 [24]	Retrospective, multi-center (including Kuerer et al., 2018 [22] and Lee et al., 2020 [23])	Ultrasound- or mammography-guided VAB, CNB or FNA	n = 166	19% (95% CI, 11–29%)	3.2% (if lesion on post-NST imaging <2 cm, and ≥6 biopsy cores; n = 76)
Sutton et al., 2021	Prospective, single center	MRI-guided VAB	n = 20	14% (95% CI, 0–58%)	NA
Pfob et al., 2021 [19]	Retrospective, multi-center (including Kuerer et al., 2018 [22], Lee et al., 2020 [23], and Heil et al., 2020 [18])	Intelligent VAB (Artificial Intelligence algorithm)	n = 507 (457 for algorithm development and testing, 50 for validation)	0% (95% CI, 0–13%)	NA

CI = confidence interval; NST = neoadjuvant systemic treatment; VAB = vacuum-assisted biopsy; FNA = fine needle aspiration; CNB = core needle biopsy.

## 4 Omitting breast and axillary surgery altogether

While the surgical management of the primary tumor in the breast and in the axilla has been mainly considered independent during the past decades, more recent evidence suggests, that axillary lymph node metastasis are mainly driven by the biology of the primary tumor [17]. As the concept of an intelligent VAB––an artificial intelligence algorithm that uses contextualizing imaging, patient, and tumor variables in addition to the results of a minimally invasive biopsy after NST––showed great potential to exclude residual disease in the breast (ypT0) [19], it has subsequently been evaluated to identify breast cancer patients with a pCR (ypT0 and ypN0) after NST. In the external validation set, the intelligent VAB showed a promising false-negative rate of 0% and a specificity of 40% to exclude residual disease in the breast or axilla [26]. Given validation in confirmatory trials, the omission of breast and axillary surgery may be evaluated for these patients in future trials.

## 5 Conclusion

Breast and axillary surgery after NST for women with breast cancer has undergone multiple paradigm changes within the past years. For axillary disease, ALND, SLNB, or TAD are nowadays recommended depending on the lymph node status before and after NST. For the primary tumor in the breast, breast conserving surgery remains the standard of care. The clinical management of exceptional responders to NST is a pressing knowledge gap due to the increasing number of patients who achieve a pathologic complete response to NST and for whom surgery may have no therapeutic benefit. Current clinical research evaluates whether less invasive procedures can exclude residual cancer as reliably as surgery to possibly omit surgery for those patients in the future.

## Funding

There was no funding for this article.

## Ethical approval

Not required.

## Declaration of competing interest

None.
